# Dual Drug Loaded Biodegradable Nanofibrous Microsphere for Improving Anti-Colon Cancer Activity

**DOI:** 10.1038/srep28373

**Published:** 2016-06-21

**Authors:** Rangrang Fan, Xiaoling Li, Jiaojiao Deng, Xiang Gao, Liangxue Zhou, Yu Zheng, Aiping Tong, Xiaoning Zhang, Chao You, Gang Guo

**Affiliations:** 1 State Key Laboratory of Biotherapy and Cancer Center, and Department of Neurosurgery, West China Hospital, Sichuan University, and Collaborative Innovation Center for Biotherapy, Chengdu 610041, P. R. China; 2 Department of Pharmacology and Pharmaceutical Sciences, School of Medicine, Tsinghua University, and Collaborative Innovation Center for Biotherapy, Beijing 100084, P. R. China

## Abstract

One of the approaches being explored to increase antitumor activity of chemotherapeutics is to inject drug-loaded microspheres locally to specific anatomic sites, providing for a slow, long term release of a chemotherapeutic while minimizing systemic exposure. However, the used clinically drug carriers available at present have limitations, such as their low stability, renal clearance and residual surfactant. Here, we report docetaxel (DOC) and curcumin (CUR) loaded nanofibrous microspheres (DOC + CUR/nanofibrous microspheres), self-assembled from biodegradable PLA-PEO-PPO-PEO-PLA polymers as an injectable drug carrier without adding surfactant during the emulsification process. The obtained nanofibrous microspheres are composed entirely of nanofibers and have an open hole on the shell without the assistance of a template. It was shown that these DOC + CUR/nanofibrous microspheres could release curcumin and docetaxel slowly *in vitro*. The slow, sustained release of curcumin and docetaxel *in vivo* may help maintain local concentrations of active drug. The mechanism by which DOC + CUR/nanofibrous microspheres inhibit colorectal peritoneal carcinomatosis might involve increased induction of apoptosis in tumor cells and inhibition of tumor angiogenesis. *In vitro* and *in vivo* evaluations demonstrated efficacious synergistic antitumor effects against CT26 of curcumin and docetaxel combined nanofibrous microspheres. In conclusion, the dual drug loaded nanofibrous microspheres were considered potentially useful for treating abdominal metastases of colorectal cancer.

Approximately 60% of cases and 70% of deaths occur in individuals newly diagnosed with colorectal cancer aged 65 years and older in 2014^1^,^2^. Colorectal peritoneal carcinomatosis is considered generally to be extremely difficult to cure effectively, with few treatment options beyond palliative care^3^,^4^. The conventional treatment approach for patients with colorectal peritoneal carcinomatosis is systemic chemotherapy; however, the median survival time of the patients is less than 12 months^5^. Because colorectal peritoneal carcinomatosis is a form of locoregional cancer dissemination, patients with this condition should be given a local treatment^6^.

In the development of suitable carriers for the locoregional delivery of anticancer drugs, micro- and nanoparticles have been pursued to achieve satisfactory results^7^,^8^,^9^,^10^,^11^. Microspheres can deliver drugs locally to the treatment site with increasing anticancer potency while reducing side effects. In addition, microspheres can circumvent challenges associated with systemic chemotherapy, such as renal clearance and degradation by serum nucleases. Researchers have found that the size and shape of the carriers strongly affect their blood circulation times and transport through different biological barriers^12^,^13^. Tsai *et al.* studied the effect of drug carrier on the antitumor activity of intraperitoneal chemotherapy, and the results showed that the microparticles had much longer residence time, greater peritoneal targeting advantage and longer survival extension compared to the nanoparticle formulations^14^. Because of this, we chose microspheres to deliver the chemotherapy. The size, shape and porosity are important structures of a drug delivery system, among these, the porosity and size of the microspheres are important for drug release kinetics and their degradation behavior^15^,^16^.

Docetaxel (DOC), a semisynthetic taxane analog derived from the needles of the European yew tree Taxus baccarat, is approved for the treatment of various types of cancer^17^. Its cytotoxic properties allow it to inhibit microtubule depolymerization, mitosis, cell cycle progression and promote tubulin assembly^18^,^19^. Like other cytotoxic agents, the actions of DOC to prevent new cell formation and induce apoptosis are not specifically for the tumor cells; ‘healthy’ cells may be adversely affected as well^20^. Hence, chemotherapeutics sometimes lead to severe toxicity at their therapeutic doses^21^.

Combination therapy with different drugs has played very important roles in the treatment of cancers to achieve higher antitumor efficacy and minimize the resistance. A number of natural herb-containing combination remedies have been reported to reduce the required drug dose^22^. Chen *et al.* found that the addition of thalidomide to docetaxel contributed to better survival prognosis than docetaxel alone^23^. Park *et al.* discovered that combination of capecitabine and docetaxel is highly active in patients with previously untreated advanced gastric carcinoma^24^.

The polyphenol curcumin (CUR), a yellow pigment of the rhizome of the plant *Curcuma longa*, is a major component of the Indian spice turmeric. Previous studies have reported that CUR exhibits a wide spectrum of therapeutic properties such as anti-carcinogenic, antioxidant, antiulcer, antimicrobial and anti-inflammatory properties^25^,^26^. Evidence has shown that it is pharmacologically safe even at high doses^27^,^28^. Increasing evidence suggested that CUR induced cell cycle arrest to tumor cells at the G2/M phase and inhibited cell proliferation by inducing apoptosis^29^,^30^. It has been used against various types of cancers, including colon cancer, with little or no toxicity^31^. Lin *et al.* indicated that curcumin inhibited the signal transducers and activators of transcription (STAT 3) phosphorylation, cell viability, and tumoursphere-forming capacity of the ALDH + /CD133+ subpopulation from colorectal cancer cells^32^. In addition, Sreekanth *et al.* observed that curcumin could repress NF-κB-dependent gene products, inhibit constitutively active in 3-methylcholanthrene-induced cervical tumorigenesis and enhance the cytotoxic effect of paclitaxel; the combinatorial treatment of paclitaxel and curcumin synergisticly reduced tumor volume, and enhanced apoptosis in xenograft tumors compared with individual treatments^33^. Based on this we formulated the hypothesis that coadministration of DOC and CUR in a single formulation may enhance the therapeutic efficacy of DOC in CT26 cells.

In the present study, a PLA-PEO-PPO-PEO-PLA (PLA-Pluronic F68-PLA, PLFL) block copolymer was successfully prepared from L-lactide and Pluronic^®^ F68 using a ring-opening polymerization method. Pluronic, poly(ethyleneoxide)–poly(propylene oxide)–poly(ethylene oxide) (PEO–PPO–PEO), a water-soluble biocompatible polymer, has been widely used to design drug delivery systems^34^,^35^,^36^. Additionally, researchers suggested that PEO had higher affinity to the surface of the small intestine^37^,^38^. Surfactant-free nanofibrous microspheres were self-assembled from PLFL block copolymer. Encapsulation of both DOC and CUR in the PLFL nanofibrous microspheres improves the solubility and stability of both hydrophobic drugs.

## Results and Discussion

Synergism of multiple drugs is a more effective strategy in the treatment of cancer because it avoids undesirable toxicity and provides synergistic therapeutic effects. A number of docetaxel-containing combination methods have been proposed to minimize the amount of drug necessary, reduce toxicity and achieve synergistic therapeutic effect in cancer treatment^18^,^19^. However, intravenous injection and oral ingestion, conventional methods for chemotherapeutic agent delivery, fail to achieve therapeutic concentrations of chemotherapeutic agents at tumor sites. Biodegradable polymeric microspheres as a controlled drug delivery system for antitumor therapy based on peritoneal injection to tumor sites have longer retention times and higher peritoneal concentrations without drug aggregation.

Microspheres and nanospheres are often used to package therapeutic cargo such as growth factors, gene, proteins, cytotoxic agent or living cells in drug delivery system^15^,^39^. However, traditional microsphere fabrication techniques such as emulsification and emulsion polymerization often require the addition of a surfactant, which may change the biodistribution, biodegradability and drug release behavior of the drug loaded microspheres^40^,^41^,^42^. Thermally induced phase separation (TIPS) has been widely used for fabrication of various scaffolds^43^. In this study, a novel rapid cooling TIPS process was developed to fabricate injectable nanofibrous microspheres without adding surfactant during the emulsification process. The PLFL solution was emulsified into liquid microspheres in glycerol. The mixture was quenched in liquid nitrogen to induce phase separation, and microspheres with open holes on the nanofibrous shells were obtained after the removal of the solvent via extraction with distilled water and then freeze-drying. Compared to the nanofibrous sheet, the nanofibrous microspheres prepared in our manuscript are injectable scaffolds, which can be advantageous in delivering bioactive agents, allowing for minimally invasive procedures to minimize complications and pain. The schematic illustration of DOC + CUR/nanofibrous microspheres and the improved anti-colon cancer activity were shown in Supplementary Fig. S1.

### Design, self-assembly, and characterization of nanofibrous microspheres

**Figure 1:**
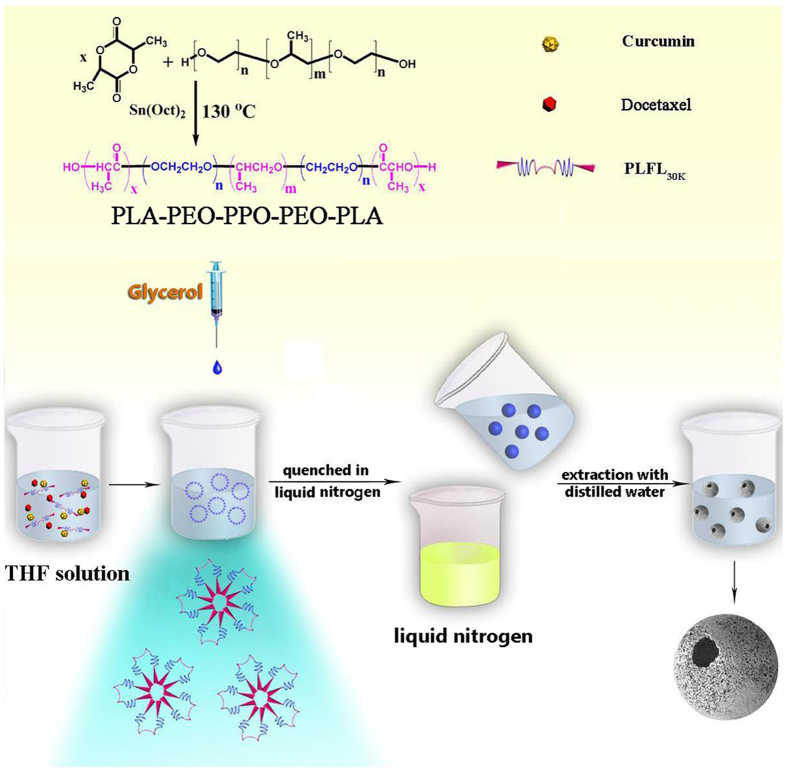
The synthesis scheme of PLFL and the schematic illustration of synthesis of DOC + CUR/nanofibrous microspheres. The figure was drawn with ChemDraw and Adobe Photoshop by the author R.R.F.

Pluronic^®^ F68, consisting of hydrophilic PEO and hydrophobic PPO blocks, was used in the study because of its commercial availability, biocompatibility and safety^44^. Researchers have reported that Pluronic block copolymers could sensitize resistant cancer cell lines, resulting in an increase in the cytotoxic activity of the drug by 2–3 orders of magnitude. It has also been reported that the copolymer could also reduce multidrug resistance (MDR), reducing its P-glycoprotein (Pgp) mediated efflux from cells^45^,^46^. As illustrated in the synthesis scheme of PLA-PEO-PPO-PEO-PLA (PLA-Pluronic F68-PLA, PLFL) (Fig. 1), the PLFL block copolymers were synthesized from L-lactide and F68 by ring-opening polymerization. ^1^H-NMR spectra of the series PLFL were recorded (Supplementary Fig. S2). And the GPC curves of prepared PLFL block copolymers were shown in Supplementary Fig. S3. Macromolecular weight and macromolecular weight distribution (dispersity, *M*_w_/*M*_n_) were summarized in Table 1. According to Table 1, the macromolecular weight (*M*_n_) estimated from ^1^H-NMR spectra were consistent with theoretical value calculated from feed ratio, which indicated that the PLFL block copolymers were prepared successfully with a controlled macromolecular weight. So, for simplicity, the feed ratio was used in the following text instead of the experimental composition ratio calculated from the ^1^H-NMR spectra.

TG curves of PLFL_20K_, PLFL_30K_, PLFL_45K_ (Supplementary Fig. S4) indicated that the peaks of thermodegradation shifted to higher temperatures and decreased in height with the increase of the *M*_n_ of PLFL. The two-stage degradation pattern suggested that the structure of PLFL block copolymers could be partially blocked. In the first stage weight loss started at about 220 °C, with the maximum being at about 280 °C, which is the characteristic thermal degradation behavior of PLLA^47^. The second degradation peak should be essentially due to that of Pluronic blocks. These results indicate that PLFL block copolymers copolymers were synthesized successfully.

Hemolytic tests were performed on the PLFL nanofibrous microspheres (Supplementary Fig. S5), PLFL nanofibrous microspheres at the concentration of 40 mg mL^−1^ did not cause any hemolysis to rabbit erythrocytes compared to the negative control (normal saline). Meanwhile, the cytotoxicity of blank PLFL nanofibrous microspheres was evaluated using a cell viability assay on L929 cells. According to Supplementary Fig. S6, cell proliferation was not suppressed by blank PLFL nanofibrous microspheres (2,400 μg mL^−1^) *in vitro*, implying non-toxicity.

**Figure 2:**
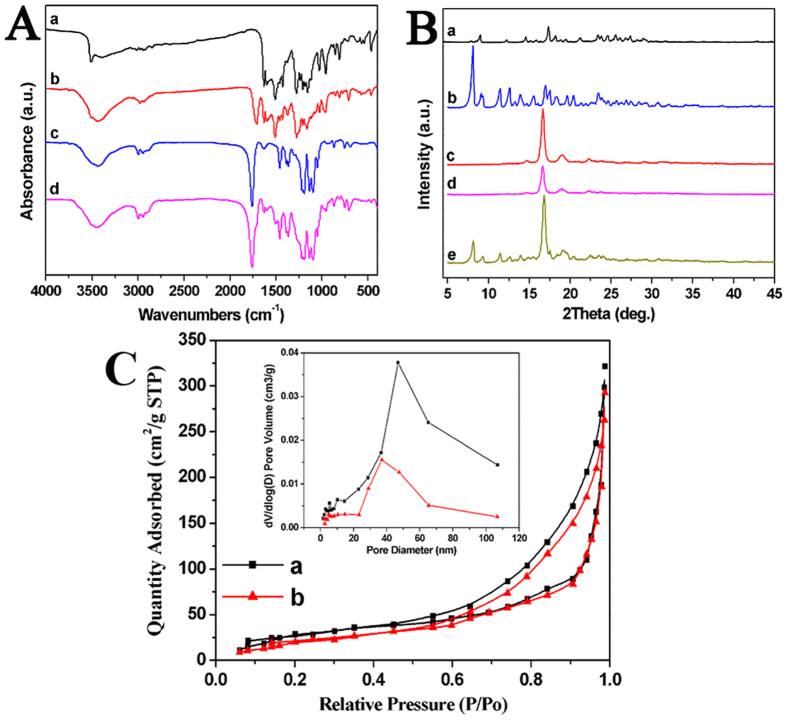
FT-IR, XRD and BET assay. (**A**) FT-IR spectra of free CUR (a), free DOC + CUR (b), blank nanofibrous microspheres (c), DOC + CUR/nanofibrous microspheres (d). (**B**) XRD patterns of free CUR (a), free DOC + CUR (b); blank nanofibrous microspheres (c), DOC + CUR/nanofibrous microspheres (d), mixture of blank nanofibrous microspheres/free DOC + CUR (e). (**C**) The nitrogen adsorption/desorption isotherms and the pore size distribution plots of the prepared nanofibrous microspheres, blank nanofibrous microspheres (a); DOC + CUR/nanofibrous microspheres (b).

Nanofibrous microspheres were prepared using a special self-assembling method. Drug loading efficiency and microsphere sizes were listed in Table 2. In consideration of particle size and drug loading, MS-3 was used in the *in vitro* and in *vivo* studies. The new bands of DOC + CUR/nanofibrous microspheres at approximately 480 cm^−1^, 1,000 cm^−1^ and 1,520 cm^−1^ (Fig. 2A) compared to the spectra blank nanofibrous microspheres can also be observed in the spectra of free CUR and free DOC + CUR, certifying that DOC and CUR were encapsulated successfully. And according to Fig. 2B, pure DOC is crystalline, with characteristic peaks at 2θ = 8.0°, 9.2°, 11.3°, 12.5°, 13.8°, and 16.9°. Pure CUR is also crystalline, with characteristic peaks at 2θ = 8.9°, 17.4°, 23.4°, and 25.6°. When comparing XRD diagrams of free DOC + CUR, blank MS and physical mixtures of blank microspheres and free DOC + CUR freeze-dried powder, the absence of specific diffraction peaks in the DOC + CUR/nanofibrous microspheres diagram indicated that DOC and CUR were encapsulated amorphously. ^1^H-NMR spectra of DOC + CUR/nanofibrous microspheres were recorded (Supplementary Fig. S7). Compared to the ^1^H-NMR spectra of DOC (Supplementary Fig. S7) and PLFL (Supplementary Fig. S2), the peaks at 1.25 ~ 1.35 and 4.25 ~ 4.40 in Supplementary Fig. S7 (C) indicated that DOC was encapsulated successfully; and the peaks at 3.75 ~ 4.00 and 6.90 ~ 7.00 in Supplementary Fig. S7 (C) indicated that CUR was encapsulated successfully. The peaks at 7.30 ~ 8.10 also indicated that DOC and CUR were encapsulated successfully.

**Figure 3:**
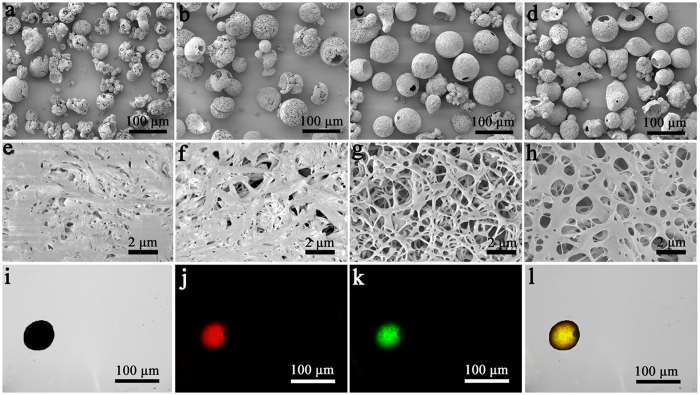
SEM images of nanofibrous microspheres. Nanofibrous microspheres made from PLFL_10K_ (**a**,**e**), PLFL_20K_ (**b**,**f**), PLFL_30K_ (**c**,**g**), PLFL_45K_ (**d**,**h**) (**a**–**d** ×200, **e**–**h** ×5,000). And morphology studies of prepared rhodamine B-coumarin 6/nanofibrous microspheres. Bright field of nanofibrous microspheres (**i**), rhodamine B fluorescence field of nanofibrous microspheres (**j**), Coumarin 6 fluorescence field of nanofibrous microspheres (**k**), merge field of rhodamine B-coumarin 6/nanofibrous microspheres (**l**).

The nitrogen adsorption/desorption isotherms and the pore size distribution plots of the prepared nanofibrous microspheres are shown in Fig. 2C, and the BET surface area of the nanofibrous microspheres was about 132 ± 1.25 m^2^/g. According to the inset in Fig. 2C, the pore-size distribution showed a peak mesopore diameter of 47 nm along with macropores up to 110 nm in size. The SEM micrographs of the nanofibrous microspheres obtained are shown in Fig. 3(a–h). The nanofibrous microspheres have an open and hollow structure. Their surfaces are composed entirely of nanofibers with an average diameter of 670 ± 67 nm (Fig. 3g). The surface morphology of nanofibrous microspheres made from PLFL_10K_ and PLFL_20K_ had an irregular shape and disorganized nanofibers. The reason may be that lower molecular weight is not conducive to balling. The surface morphology of PLFL_45K_ nanofibrous microspheres was superior to PLFL_10K_ and PLFL_20K_ nanofibrous microspheres; however, they were inferior to PLFL_30K_ nanofibrous microspheres. This may be because PLFL_45K_ is adverse to molecular self-folding. Because of this, PLFL_30K_ was used in the following studies. Under fluorescent microscopy, we found that rhodamine B- and coumarin 6-loaded nanofibrous microspheres were spherical with a diameter of approximately 70 μm. Rhodamine B (red fluorescence, Fig. 3j) and coumarin 6 (green fluorescence, Fig. 3k) were encapsulated in the nanofibrous microspheres.

**Figure 4:**
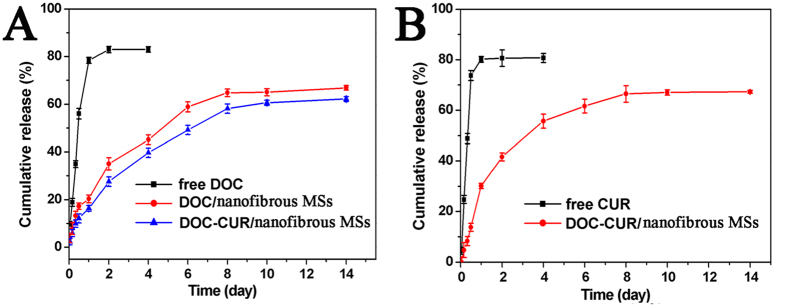
*In vitro* release behavior of DOC and CUR. (**A**) *In vitro* release behavior of DOC from free DOC, DOC/nanofibrous microspheres and DOC + CUR/nanofibrous microspheres. (**B**) *In vitro* release behavior of CUR from free CUR and DOC + CUR/nanofibrous microspheres. Error bars represent the SD (n = 3).

Figure 4A showed the release profiles of DOC from free DOC, DOC/nanofibrous microspheres and DOC + CUR/nanofibrous microspheres in PBS (pH 7.4, 37 °C); Fig. 4B showed the release profiles of CUR from free CUR and DOC + CUR/nanofibrous microspheres. Compared to free DOC and CUR, typical two-phase-release profiles of DOC/nanofibrous microspheres and DOC + CUR/nanofibrous microspheres showed relatively rapid release in the first stage followed by a sustained and slow release over a prolonged period up to several weeks. It was found that only 18% DOC was released from DOC/nanofibrous microspheres, and 15% DOC, 21% CUR were released from DOC + CUR/nanofibrous microspheres within 24 h. In contrast, free DOC and free CUR released approximately 80% into the outside media within 24 h. This confirms that the nanofibrous microspheres provided for a slow, long term release of a chemotherapeutic.

### Enhanced *in vitro* antitumor and cell apoptosis activity

The toxicity of free DOC, free CUR, and free DOC + CUR (2:1, 1:1, 1:2) containing equivalent concentrations of DOC was evaluated using MTT assay (Supplementary Fig. S8, In Supplementary Fig. S8 (C–E), the horizontal ordinate refers to the doses of DOC). All samples at various concentrations significantly decreased the viability of CT26 cells in a dose-dependent manner. As shown in Table 3, half maximal inhibitory concentration (IC50) of free DOC + CUR (1:2) was 1.41 ± 0.16 μg mL^−1^, lower than that of free DOC + CUR (1:1) (1.44 ± 0.23 μg mL^−1^), free DOC + CUR (2:1) (1.68 ± 0.19 μg mL^−1^), free DOC (2.2 ± 0.21 μg mL^−1^) and free CUR (14.97 ± 1.24 μg mL^−1^). This suggested that CUR in combination with DOC could achieve higher antitumor efficacy. The antitumor effect of free DOC + CUR (1:1) was similar to that of free DOC + CUR (1:2). In light of this, we chose a 1:1 ratio as our study object in the preparation of drug-loaded microspheres.

The DOC and CUR concentrations to inhibit CT26 proliferation when used alone or in combination (2:1, 1:1, 1:2) are listed in Supplementary Table S1, Supplementary Table S2 and Supplementary Table S3, and the combination index was also listed. As shown in these Tables, the presence of both agents, the doses of DOC and CUR required to achieve inhibition of 50, 70 and 90% of CT26 proliferation were considerably reduced. In Supplementary Table S1 and Supplementary Table S2 the combination of DOC/CUR demonstrates an additive effect (CI > 1) to produce 10% and 30% inhibition of CT26 proliferation and synergism (CI < 1) to achieve 50, 70, and 90% of CT26 proliferation. In Supplementary Table S3, the combination of DOC/CUR demonstrates an additive effect (CI > 1) to produce 10%, and 30% inhibition of CT26 proliferation and synergism (CI < 1) to achieve 50%, 70% and 90% of CT26 proliferation, however, the CI was close to 1 when the CT26 proliferation was 50% and 90%. CI was plotted as a function of the fraction affected (fa) (Supplementary Fig. S9a), which represents the percentage of growth inhibition, evaluated using the MTT assay (0.5 = 50%).

**Figure 5:**
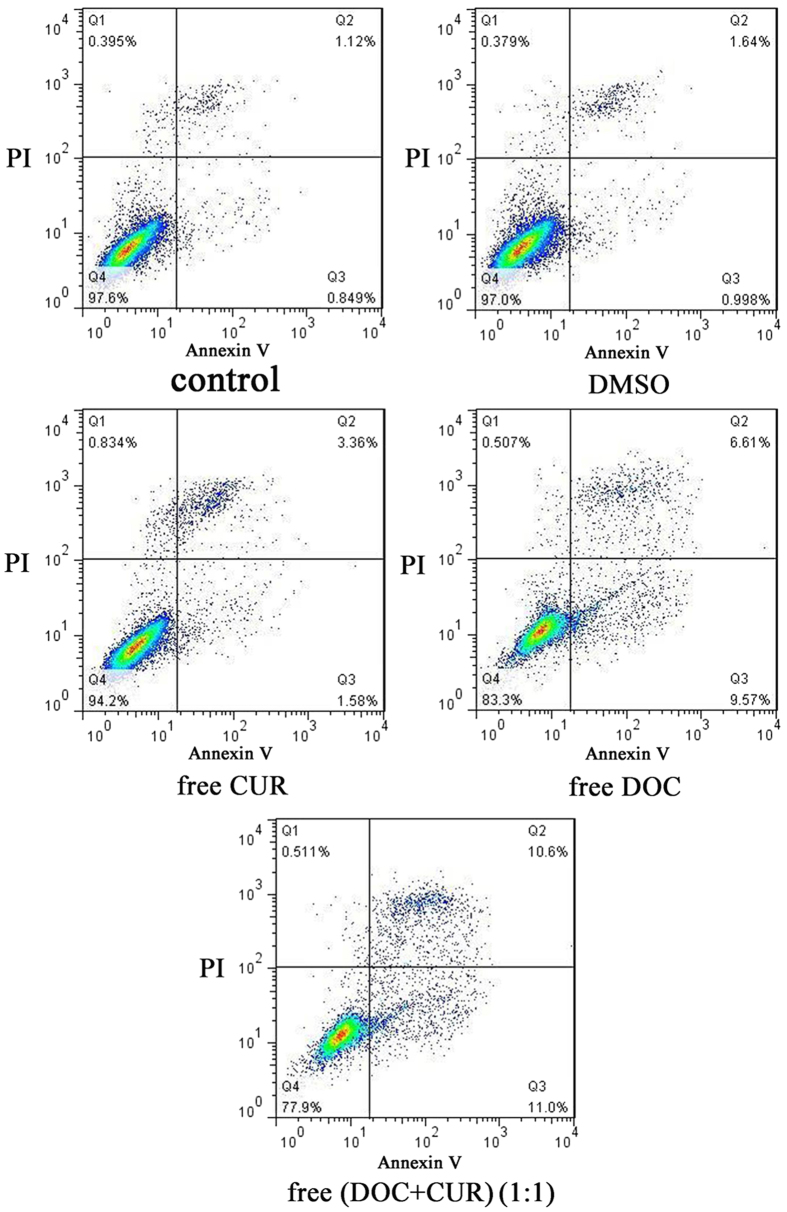
The apoptosis in CT26 cells of free Cur, free DOC and free DOC + CUR (1:1). The CT26 cells were incubated with free CUR (2 μg mL^−1^), free DOC (2 μg mL^−1^) and free DOC + CUR (1:1) (2 μg mL^−1^: 2 μg mL^−1^) for 12 h, and Annexin V and PI-stained cells were determined by flow cytometry. Independent experiments were repeated three times with similar results.

Flow cytometry analysis was used to investigate apoptosis induction of free DOC, free CUR, and free DOC + CUR (1:1) containing equivalent concentrations of DOC (2 μg mL^−1^) in CT26 cells by observing apoptotic cells. As shown in Fig. 5, the percentage of Annexin V-positive cells was 4.9%, 16.2%, and 21.6% in the free CUR, free DOC and free DOC + CUR (1:1) treatment groups, respectively. The control and DMSO groups did not show apoptosis as observed in Fig. 5. Compared to free CUR and free DOC, the free DOC + CUR group showed enhanced apoptosis. It was clearly evident that combination therapy of DOC and CUR has a synergistic therapeutic effect in treating colorectal peritoneal carcinomatosis.

### *In vivo* antitumor activity

**Figure 6:**
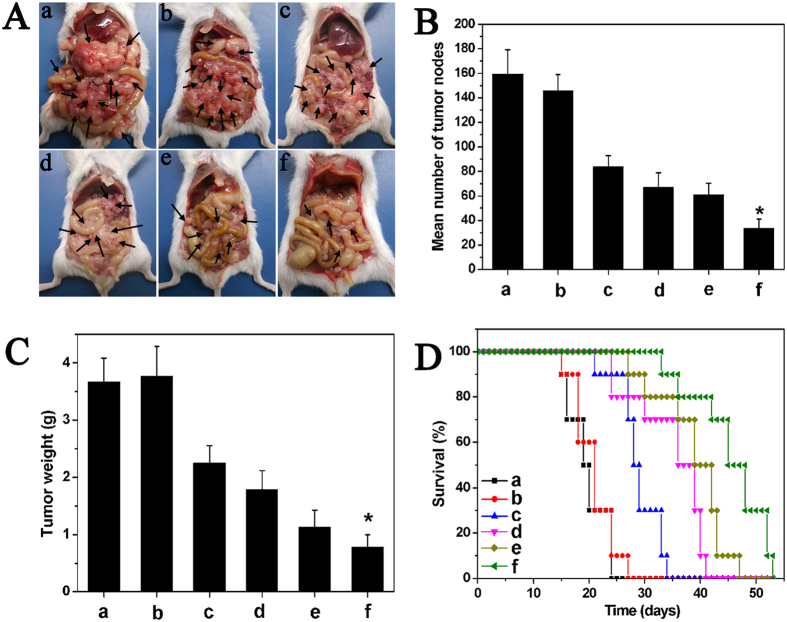
Inraperitoneal administration of DOC + CUR/nanofibrous microspheres inhibited the growth of abdominal metastases of CT26 colon carcinoma. (**A**) Representative photographs of tumor nodules (arrows) in each group. (**B**) Number of tumor nodules in each group. The results were expressed as average ± SD (n = 6). (**C**) Weight of tumor nodules in each group. The results were expressed as average ± SD (n = 6). (**D**) Survival curve of mice in each group. In this Figure, NS (a), blank nanofibrous microspheres (b), free DOC (c), DOC/nanofibrous microspheres (d), free DOC + CUR (e), and DOC + CUR/nanofibrous microspheres group (f), respectively. NS is for normal saline group as control.

The anticancer activity of the intraperitoneal injection of drug-loaded nanofibrous microspheres in Balb/c mice with CT26 colon carcinoma was illustrated in Fig. 6. The abdominal cavity images showed that the tumor node numbers from the DOC + CUR/nanofibrous microspheres treated group were significantly lower than those of the other groups. Furthermore, the size of the tumor nodes was significantly smaller than those of the other groups. The *in vivo* survival rates of each group were also examined (Fig. 6D). After administration of saline and blank nanofibrous microspheres, all mice died within 27 days because of the rapid growth of the tumors. The median survival times of the saline and blank nanofibrous microsphere groups were 18 and 20 days, respectively. The median survival time in the DOC + CUR/nanofibrous microspheres group (48 days) was significantly longer than that in the free DOC + CUR (42 days, P < 0.05), DOC/nanofibrous microspheres (39 days, P < 0.05), free DOC (29 days, P < 0.05), blank nanofibrous microspheres (20 days, P < 0.05), and NS (18 days, P < 0.05) groups.

**Figure 7:**
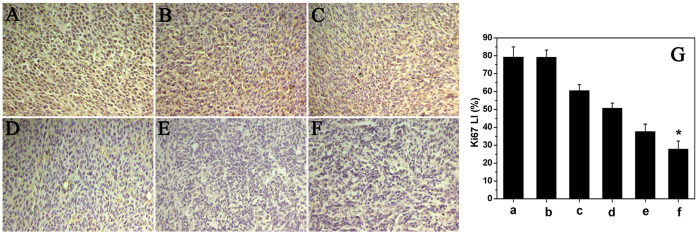
Ki67 immunofluorescent staining of tumors. Representative ki67 immunofluorescent images of NS (**A**), blank nanofibrous microspheres (**B**), free DOC (**C**), DOC/nanofibrous microspheres (**D**), free DOC + CUR (**E**), DOC + CUR/nanofibrous microspheres group (**F**), and mean Ki-67 LI in each group (**G**) (a–f corresponding to (**A**–**F**), respectively). Error bars represent the SD (n = 6). NS is for normal saline group as control.

**Figure 8:**
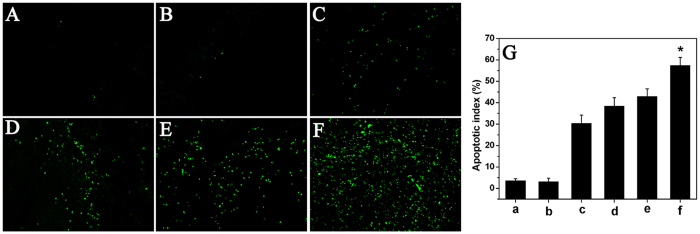
TUNEL immunofluorescent staining of tumors. Representative TUNEL immunofluorescent images of NS (**A**), blank nanofibrous microspheres (**B**), free DOC (**C**), DOC/nanofibrous microspheres (**D**), free DOC + CUR (**E**), DOC + CUR/nanofibrous microspheres group (**F**), and mean apoptotic index in each group (**G**) (a–f corresponding to (**A–F**), respectively). Error bars represent the SD (n = 6). NS is for normal saline group as control.

To evaluate the effect of combination therapy microspheres on tumor proliferation, immunohistochemical staining murine Ki-67 was carried out. As shown in Fig. 7, within a similar high-power field, weak Ki-67 immunoreactivity in tumor tissues were observed in DOC + CUR/nanofibrous microspheres treated mice compared to those in other groups. The Ki-67 LI of tumor tissues was significantly lower in the DOC + CUR/nanofibrous microspheres group (27.83 ± 4.57%) than in the free DOC + CUR (37.71 ± 4.12%, P < 0.001), DOC/nanofibrous microspheres (50.67 ± 2.79%), free DOC (60.47 ± 3.40%), blank nanofibrous microspheres (79.15 ± 4.07%, P < 0.001), or NS groups (79.21 ± 5.78%, P < 0.001) (Fig. 8G). These results indicated that combination therapy microspheres could suppress tumor cell proliferation.

To study the mechanism associated with the anticancer activity of DOC + CUR/nanofibrous microspheres *in vivo*, a TUNEL assay was carried out. As shown in Fig. 8, many strongly positive nuclei identified as apoptotic could be observed in the DOC + CUR/nanofibrous microspheres treated tumor tissues, whereas such nuclei were sparser in other groups. The apoptotic index in the DOC + CUR/nanofibrous microspheres group was 57.33 ± 3.83%, versus 42.83 ± 3.65% in the free DOC + CUR group (P < 0.01), 38.33 ± 3.93% (P < 0.01) in the DOC/nanofibrous microspheres group, 30.33 ± 3.83% (P < 0.01) in the free DOC group, 3.16 ± 1.60% (P < 0.01) in the blank nanofibrous microspheres group and 3.50 ± 1.05% (P < 0.01) in the NS group. This implies that apoptosis induction may be one mechanism that DOC + CUR combination therapy uses to inhibit colon cancer *in vivo*.

**Figure 9:**
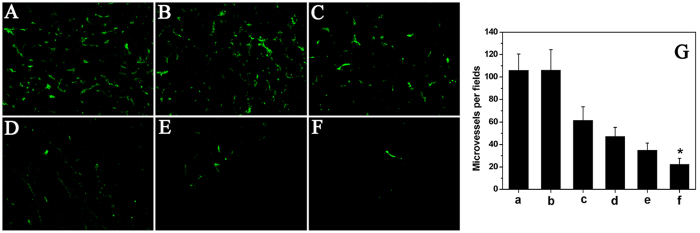
CD31 immunofluorescent staining of tumors. Representative CD31 immunofluorescent images of NS (**A**), blank nanofibrous microspheres (**B**), free DOC (**C**), DOC/nanofibrous microspheres (**D**), free DOC + CUR (**E**), DOC + CUR/nanofibrous microspheres group (**F**), and MVD in each group (**G**) (a–f corresponding to (**A–F**), respectively). Error bars represent the SD (n = 6). NS is for normal saline group as control.

Tumor sections of each treatment group were stained with CD31 for evaluation of the microvessel density (MVD). The combined nanofibrous microspheres treatment resulted in dramatic inhibition of angiogenesis in the tumors (Fig. 9). The MVD in the DOC + CUR/nanofibrous microspheres group was 22.17 ± 5.49, which was dramatically lower than that in the free DOC + CUR group (34.91 ± 6.40, P < 0.01), DOC/nanofibrous microspheres group (47.10 ± 8.19, P < 0.01), free DOC group (61.57 ± 11.83, P < 0.01), blank nanofibrous microspheres group (105.98 ± 18.41, P < 0.01) and NS (105.78 ± 14.79, P < 0.01) group. The results implied that anti-angiogenesis may be another mechanism of inhibiting colon cancer by the DOC + CUR/nanofibrous microspheres *in vivo*.

## Materials and Methods

### Materials, cell lines and animals

PEO–PPO–PEO triblock copolymer Pluronic® F68 (PEO–PPO–PEO triblock copolymer) and L-lactide were purchased from BASF (Germany) and Guangshui National Chemical Co., Ltd. (China), respectively. Curcumin (Sigma-Aldrich, USA), Coumarin 6 (Sigma-Aldrich, USA), 3-[4,5-dimethylthiazol-2-yl]-2,5-diphenyltetrazolium bromide (MTT, Sigma-Aldrich, USA) and Stannous octanoate (Sn(Oct)2, Sigma-Aldrich, USA), Docetaxel (Sichuan Xieli Pharmaceutical Co., Ltd., chengdu, China) and Rhodamine B (Ke Long chemicals, chengdu, China) were used without further purification. All other chemicals used in this work were of analytical reagent (AR) grade and used without further purification.

CT26 cells and L929 cells were obtained from the ATCC (American Type Culture Collection, USA) and grown in RPMI 1640 medium (Gibco, USA) containing 10% fetal bovine serum (FBS, Gibco, USA). The cells were cultured at 37 °C with a humidified atmosphere containing 5% CO_2_.

Balb/c mice (18–20 g) were used for *in vivo* tumor model and treat plan studies. The animals were obtained from the Animal Center Laboratory of Beijing HFK Bioscience Co., Ltd (Beijing, China, which were housed at controlled temperature (20–22 °C), relative humidity of 50–60% and 12-hour light–dark cycles. All the animals were provided with standard laboratory chow and tap water ad libitum and all the procedures were performed according to the protocol approved by the Institutional Animal Care and Treatment Committee of Sichuan University (Chengdu, People’s Republic of China). All mice were treated humanely throughout the experimental period.

### Synthesis of PLA-PEO-PPO-PEO-PLA (PLA-F68-PLA) block copolymers

Series PLA-F68-PLA biodegradable block copolymers (PLA_5800_-F68_8400_-PLA_5800_, PLFL_20K_; PLA_10800_-F68_8400_-PLA_10800_, PLFL_30K_; PLA_18300_-F68_8400_-PLA_18300_, PLFL_45K_) were synthesized by ring-opening polymerization. Briefly, a known amount of L-lactide and F68 were introduced into a dry glass ampoule under a nitrogen atmosphere, and several drops of Sn(Oct)_2_ were added. The ampoule was kept at 130 °C for ten hours with stirring slowly. Then the system was rapidly heated to 150 °C under vacuum for an additional hour. The mixture was first dissolved in methylene chloride, filtered in vacuum, and then the obtained filtrate was precipitated in excess cold petroleum ether. The obtained PLFL copolymers was dried to constant weight in vacuum and kept in air-tight bags before further use. ^1^H-NMR spectroscopy was recorded on a BRUKER AVANCE III 400 (BRUKER, Germany) at 400 MHz using deuterated chloroform (CDCl_3_) as the solvent and tetramethylsilane (TMS) as the internal reference standard. GPC (Agilent 110 HPLC, USA) was used to determine the macromolecular weight and macromolecular weight distribution of PLA-F68-PLA copolymers.

### Preparation and characterization of combination chemotherapy nanofibrous microspheres

A rapid cooling TIPS process was developed to fabricate nanofibrous microspheres. Briefly, DOC (20 mg or 10 mg) and CUR (0 mg, 10 mg or 20 mg) were dissolved in tetrahydrofuran with a corresponding amount of PLFL (total weight was 400 mg) at 50 °C with a concentration of 2.0% (wt/v). Under mechanical stirring with a rotor device (T25, IKA, Germany) at 400 rpm, glycerol (50 °C) with three times the volume of the PLFL solution was gradually added to the PLFL solution, and the stirring continued for 5 min afterward. The mixture was then quickly poured into liquid nitrogen to induce phase separation for nanofibre formation. After 10 min, a water–ice mixture (1,000 mL) was added for solvent exchange for 24 h. The spheres were sieved and washed with distilled water in excess of six times to remove any glycerol residue. The spheres were then lyophilized for three days. Rhodamine B and Coumarin 6-loaded nanofibrous microspheres were prepared using the same method. Drug-free microspheres were produced in a similar manner but without adding the drug.

We evaluated Brunauer–Emmett–Teller (BET) surface area and mesopore volumes of the samples from their nitrogen adsorption isotherms. BET specific surface areas were examined by adsorption experiments of nitrogen and the adsorption isotherm data were collected at 77 K (TRISTAR II3020, Micromeritics, USA). Prior to analysis, fibers were degassed for at least 6 h at 20 °C. The adsorption isotherms were completed with at least 0.1 g sample in the 0.01–1 relative pressure range.

SEM (JSM-7500F, JEOL, Japan) was used to investigate the surface morphology of the PLFL nanofibrous microspheres. The samples were coated with gold before observation. A fluorescent microscopy (Leica DM2500, Germany) was used to observe the rhodamine B and coumarin 6-loaded nanofibrous microspheres.

### Fourier transform infrared absorption spectra (FT-IR).

FT-IR spectra were recorded on a Nicolet 6700 FT-IR spectrometer (Thermo Scientific, Waltham, MA, USA) in a range of 400–4,000 cm^−1^, using the KBr disk method.

### X-ray diffraction assay

X-ray diffraction (XRD) measurements of all samples were performed on an X’Pert Pro MPD DY1291 (PHILIPS, Netherlands) diffractometer using graphite monochromatized Cu K radiation (λ = 0.1542 nm; 40 kV; 40 mA). The samples were measured in the 2θ range from 5° to 50° at a scanning rate of 4 ° min^−1^.

### Thermogravimetric analysis

Thermogravimetric analysis (TGA) was carried out by using a TGA Q 500 series Thermogravimetric Analyzer (TA Instrument, USA). All samples were dried in vacuum oven before the TGA test, and then were heated from room-temperature to 600 °C with a heating rate of 10 °C min^−1^.

### Hemolysis assay

The blank PLFL nanofibrous microsphere samples were weighted and immerged in saline water for 24 h. Anticoagulated rabbit whole blood (0.2 mL) was added to each sample after being equilibrated in normal saline (0.9% sodium chloride solution in water) for 30 min at 37 °C. After 10 min, 4 mL of saline water was added to each sample to stop the hemolysis. Samples were incubated for 60 min at 37 °C. Positive and negative controls were produced by adding 0.2 mL of blood to 4 mL of distilled water and saline water, respectively.

### *In vitro* cytotoxicity against CT26 colon cancer cells

The cytotoxicity of free DOC, free CUR, and free DOC + CUR (2:1, 1:1, 1:2) containing equivalent concentration of DOC on CT26 cells was measured using trypan-blue exclusion assay and MTT (Sigma Aldrich) assay. CT26 cells were plated in 96-well plates at a density of 3 × 10^3^ cells per well in 100 μl of RPMI 1640 medium and grown for 24 h. Free DOC, free (DOC:CUR = 2:1), free (DOC:CUR = 1:1) and free (DOC:CUR = 1:2) were dissolved in DMSO. Each cell was washed twice with PBS and incubated with various concentrations of drugs for 24 h. After washing the cells with PBS, 20 μl MTT (5 mg/ml) solution was added into each cell and treated for 4 h at 37 °C. Then the supernatant was fully removed, and 150 μl DMSO was added to per cell and oscillated it to solubilize, the ultraviolet absorbance was measured at 570 nm. The percentage of metabolically active cells was compared to the survival of the control group, and all data were expressed as the mean ± SD.

Combination index (CI)-isobologram equation by Chou and Talalay (1984) was used to analyze the synergistically inhibitory effect of drug combinations^48^. CI < 1 represents synergistic cytotoxicity; CI = 1 represents addictive cytotoxicity; and CI > 1 represents antagonistic cytotoxicity^49^.

### Drug loading and encapsulation efficiency

The concentration of DOC and CUR in the nanofibrous microspheres was determined by Reverse-phase High Performance Liquid Chromatography (RP-HPLC) with an Apollo C18 column (150 mm × 4.6 mm, 5 μl; Grace) at 230 nm for DOC and 420 nm for CUR. The RP-HPLC was analyzed using a mobile phase of acetonitrile and ultrapure water in the ratio of 48:52, v/v for DOC and 60:40, v/v for CUR at a flow rate of 1.0 mL min^−1^. All analyses were performed in triplicate. Drug loading content and encapsulation efficiency were obtained by the following equations:


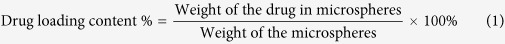



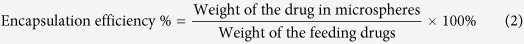


### *In vitro* release

To determine the *in vitro* release kinetics of either DOC or CUR from the nanofibrous microspheres (both single and dual drug loaded formulation), the nanofibrous microspheres in 1 mL of PBS (0.01 M, pH 7.4) containing 0.05% v/v of Tween 80 were placed in a dialysis tube (MWCO = 3,500–7,000). Then the dialysis bags were incubated with 20 mL PBS (pH = 7.4) at 37 ◦C with gentle shaking. Then incubation medium was replaced by fresh medium at specific time intervals. The concentration of DOC and CUR released from nanofibrous microspheres was determined via RP-HPLC as described previously. This study was repeated three times, and the averaged values were used in data presentation.

### Cell apoptosis assay

The extent of apoptosis in CT26 cells was evaluated via flow cytometry (ESP Elite, Beckman-Coulter, Miami, FL, USA) analysis using FITC-conjugated AnnexinV/propidium iodide (PI, BD PharMingen) staining per the manufacturer’s instructions. CT26 cells were plated at a density of 2 × 10^5^ cells per well in 2 ml 1640 growth medium in 6-well plates and incubated for the night, then free DOC, free CUR, and free DOC + CUR (1:1) containing equivalent concentrations of DOC (2 μg mL^−1^) were added into 6-well plates and incubated for 12 hours. Finally the cells were collected and determined by flow cytometric analysis with Annexin-V/PI staining. Both early apoptotic (Annexin V-positive, propidium iodide-negative) and late apoptotic (Annexin V-positive and propidium iodide-positive) cells were included in the determination of cell death.

### *In vivo* tumor model and treat plan

The antitumor effects of DOC + CUR/nanofibrous microspheres were evaluated using a colorectal peritoneal carcinomatosis (CRPC) mice model. Every mouse was intraperitoneally injected with 200 μl of cell suspension containing 2 × 10^5^ CT26 cells. Tumors were allowed to grow for 7 days. Then, tumor-bearing mice were randomly assigned to one of the following five groups (n = 12 mice per group): normal saline (NS), blank nanofibrous microspheres, free DOC (8 mg kg^−1^), free DOC:CUR mixture (1:1) (8 mg kg^−1^), and DOC + CUR/nanofibrous microspheres (8 mg kg^−1^). Treatments were administered through intraperitoneal injection with a single dose of 200 μl of the drug corresponding to the treatment group. For the tumor inhibition study (6 mice per group), the number and weight of the tumor nodules were measured on day 15, and tumor tissues were collected for immunofluorescent analysis. After day 15, the mice in the NS group began to die. To further study the therapeutic effect against colorectal cancer, the survival times of mice treated with the protocols described above were observed (10 mice per group).

### Ki-67 assay

Tumor tissue sections were fixed in 4% wt. paraformaldehyde, embedded in paraffin, and sectioned. Sections 3–5 μm thick were cut and mounted for Ki-67 staining using the labeled streptavidin-biotin method. The primary antibody and secondary antibody were rat anti-mouse monoclonal antibody Ki-67 (Gene Tech) and biotinylated goat anti-rat immunoglobulin (BD Biosciences Pharmingen), respectively. To quantify Ki-67 expression, the Ki-67 labeling index (Ki-67 LI) was calculated as number of Ki-67-positive cells/total number of cells (×400) (Leica DM2500, Germany) in five randomly selected areas in each tumor sample by two independent investigators in a blinded fashion.

### TUNEL and CD31 assays

The CT26 tumor tissues were prepared as described above for TUNEL assay. Terminal deoxynucleotidyl transferase-mediated nick-end labeling (TUNEL) staining was conducted using an *in situ* cell death detection kit (DeadEnd^TM^ Fluorometric TUNEL System, Promega, Madison, USA). This analysis was performed following the manufacturer’s protocol, and the samples were then examined using a fluorescence microscope (×400) (Leica DM2500, Germany). In each section of tumor tissue, four equal-sized fields were randomly chosen and analyzed. The apoptotic index was calculated as a ratio of the apoptotic cell number to the total tumor cell number in each high-power field.

For blood vessel staining, the tumors were stored at −80 °C to examine microvessel expression, then frozen sections of tumors were fixed in acetone, washed with PBS, stained with rat anti-mouse CD31 (platelet endothelial cell adhesion molecule-1) polyclonal antibody (1:50; BD Pharmingen^TM^, USA), washed twice with PBS, and followed by incubation with a FITC-conjugated second antibody (Abcam, USA). Microvessel density was calculated as the average number of small CD31-positive vessels in a high-power (×400) field using a fluorescence microscope (×400) (Leica DM2500, Germany).

### Statistical analysis

The statistical analysis was carried out using SPSS 15.0 software (Chicago, IL, USA). Comparisons of tumor nodules number and tumor weight were performed using one-way analysis of variance (ANOVA). Survival curves were generated based on the Kaplan–Meier method, and statistical significance was determined using Mann–Whitney U-tests. A P value < 0.05 on a 2-tailed test was considered statistically significant.

### Ethics statement

The methods were carried out in accordance with the approved guidelines. The Animal Care and Use Committee of Sichuan University (Chengdu, Sichuan, China) approved all the animal experiments.

## Additional Information

**How to cite this article**: Fan, R. *et al.* Dual Drug Loaded Biodegradable Nanofibrous Microsphere for Improving Anti-Colon Cancer Activity. *Sci. Rep.* **6**, 28373; doi: 10.1038/srep28373 (2016).

## Supplementary Material

Supplementary Information

## Figures and Tables

**Table 1:** The PLFL copolymer obtained in this paper.

Samples	*M*_n_^a^ (×10^4^)	*M*_n_^b^ (×10^4^)	Molecular weight		
			*M*_w_^c^ (×10^4^)	*M*_n_^c^ (×10^4^)	*M*_w_/*M*_n_
1	1.00	0.97	1.00	0.79	1.26
2	2.00	1.86	3.69	1.88	1.96
3	3.00	2.94	5.21	2.73	1.90
4	4.50	4.43	7.27	4.30	1.69

^a^Calculated from feed ratio.

^b^Determined by ^1^H-NMR.

^c^Determined by GPC in THF (Polystyrene standard). *M*_n_: number-average molecular weight, *M*_w_: weight-average molecular weight, polydispersity: *M*_w_/*M*_n_.

**Table 2:** The characterization of nanofibrous microspheres.

Sample	Drug/polymer DOC:CUR 1:1	DL (%) of DOC	DL (%) of CUR	EL (%) of DOC	EL (%) of CUR	Size (μm)
MS-1	2/20	1.92 ± 0.47	2.13 ± 0.35	38.98 ± 5.12	43.86 ± 4.93	42.54 ± 8.56
MS-2	2/20	2.83 ± 0.28	3.01 ± 0.34	57.15 ± 3.58	61.36 ± 2.97	68.46 ± 6.48
MS-3	2/20	3.98 ± 0.58	4.11 ± 0.29	79.84 ± 2.82	83.46 ± 3.54	73.81 ± 3.63
MS-4	2/20	3.44 ± 0.42	3.28 ± 0.31	68.27 ± 3.46	66.32 ± 4.35	71.86 ± 5.97

MS-1, MS-2, MS-3, MS-4: Nanofibrous microspheres prepared from PLFL_10K_, PLFL_20K_, PLFL_30K_ and PLFL_45K_, respectively.

**Table 3:** Half maximal inhibitory concentration (IC50) of each group.

Samples	Free CUR	Free DOC	free[DOC + CUR (2:1)]	free[DOC + CUR (1:1)]	free[DOC + CUR (1:2)]
IC50 (μg mL^−1^)	14.97 ± 1.24	2.2 ± 0.21	1.68 ± 0.19	1.44 ± 0.23	1.41 ± 0.16
