# Supplementary material for: Dual Drug Loaded Biodegradable Nanofibrous Microsphere for Improving Anti-Colon Cancer Activity
Source: Sci Rep. 2016 Jun 21;6:28373. doi: 10.1038/srep28373 (PMC4914940; doi:10.1038/srep28373)
Supplement: Supplementary Information [file srep28373-s1.pdf]

supplementary information

Dual Drug Loaded Biodegradable Nanofibrous Microsphere for Improving Anti-Colon Cancer Activity

Rangrang Fan[1,*], Xiaoling Li[1,*], Jiaojiao Deng[1], Xiang Gao[1], Liangxue Zhou[1], Yu Zheng[1], Aiping Tong[1], Xiaoning Zhang[2], Chao You[1] and Gang Guo[1]

1 State Key Laboratory of Biotherapy and Cancer Center, and Department of Neurosurgery, West China Hospital, Sichuan University, and Collaborative Innovation Center for Biotherapy, Chengdu 610041, R. P. China

2 Department of Pharmacology and Pharmaceutical Sciences, School of Medicine, Tsinghua University, and Collaborative Innovation Center for Biotherapy, Beijing 100084, P. R. China

* These authors contributed equally to this work and are co-first authors.

Correspondence and requests for materials should be addressed to G.G. (email: guogang@scu.edu.cn) or to L.X.Z. (email: liangxue_zhou@126.com).

Tel.: +86 2885164063; fax: +86 2885164060.

Supplementary Figures

Table 1 Doses necessary to inhibit CT26 proliferation in a MTT assay by 10 (ID_{10}), 30 (ID_{30}), 50 (ID_{50}), 70 (ID_{70}), 90 (ID_{90}) by single-agent docetaxel (DOC) or curcumin (CUR) and by combination of DOC and CUR (2:1).

	DOC (μg/ml)	CUR (μg/ml)	DOC/CUR (2:1) (μg/ml)	CI
ID_{10}	0.02	1	0.03/0.015	1.51
ID_{30}	0.2	8	0.2/0.1	1.01
ID_{50}	2.2	15	1.5/0.75	0.73
ID_{70}	10	26	7/3.5	0.83
ID_{90}	14	48	11/5.5	0.89

Table 2 Doses necessary to inhibit CT26 proliferation in a MTT assay by 10 (ID_{10}), 30 (ID_{30}), 50 (ID_{50}), 70 (ID_{70}), 90 (ID_{90}) by single-agent docetaxel (DOC) or curcumin (CUR) and by combination of DOC and CUR (1:1).

	DOC (μg/ml)	CUR (μg/ml)	DOC/CUR (1:1) (μg/ml)	CI
ID_{10}	0.02	1	0.025/0.025	1.28
ID_{30}	0.2	8	0.2/0.2	1.03
ID_{50}	2.2	15	1.4/1.4	0.72
ID_{70}	10	26	5/5	0.69
ID_{90}	14	48	9/9	0.83

Table 3 Doses necessary to inhibit CT26 proliferation in a MTT assay by 10 (ID_{10}), 30 (ID_{30}), 50 (ID_{50}), 70 (ID_{70}), 90 (ID_{90}) by single-agent docetaxel (DOC) or curcumin (CUR) and by combination of DOC and CUR (1:2).

	DOC (μg/ml)	CUR (μg/ml)	DOC/CUR (1:2) (μg/ml)	CI
ID_{10}	0.02	1	0.025/0.05	1.3
ID_{30}	0.2	8	0.2/0.4	1.05
ID_{50}	2.2	15	1.7/3.4	0.98
ID_{70}	10	26	4/8	0.71
ID_{90}	14	48	8/16	0.90

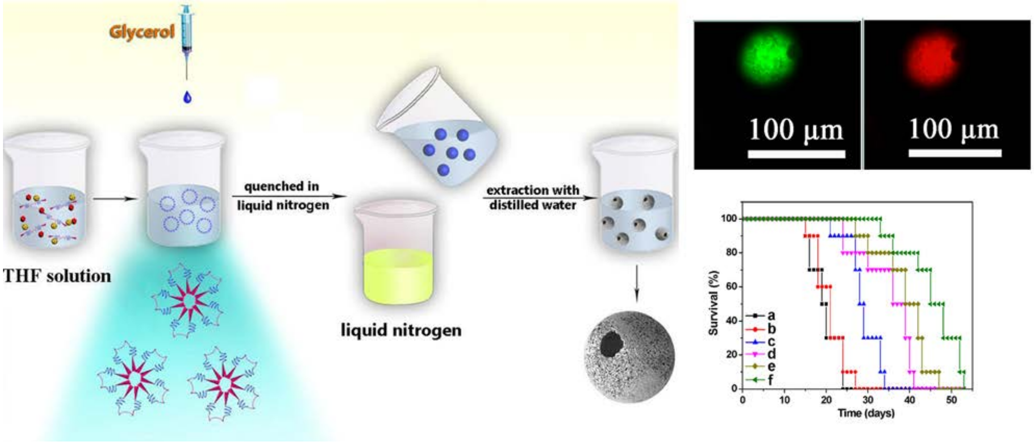

Figure S1. The schematic illustration of DOC+CUR/nanofibrous microspheres and the improved anti-colon cancer activity. The figure was drawn with ChemDraw and Adobe Photoshop by the author R.R.F.

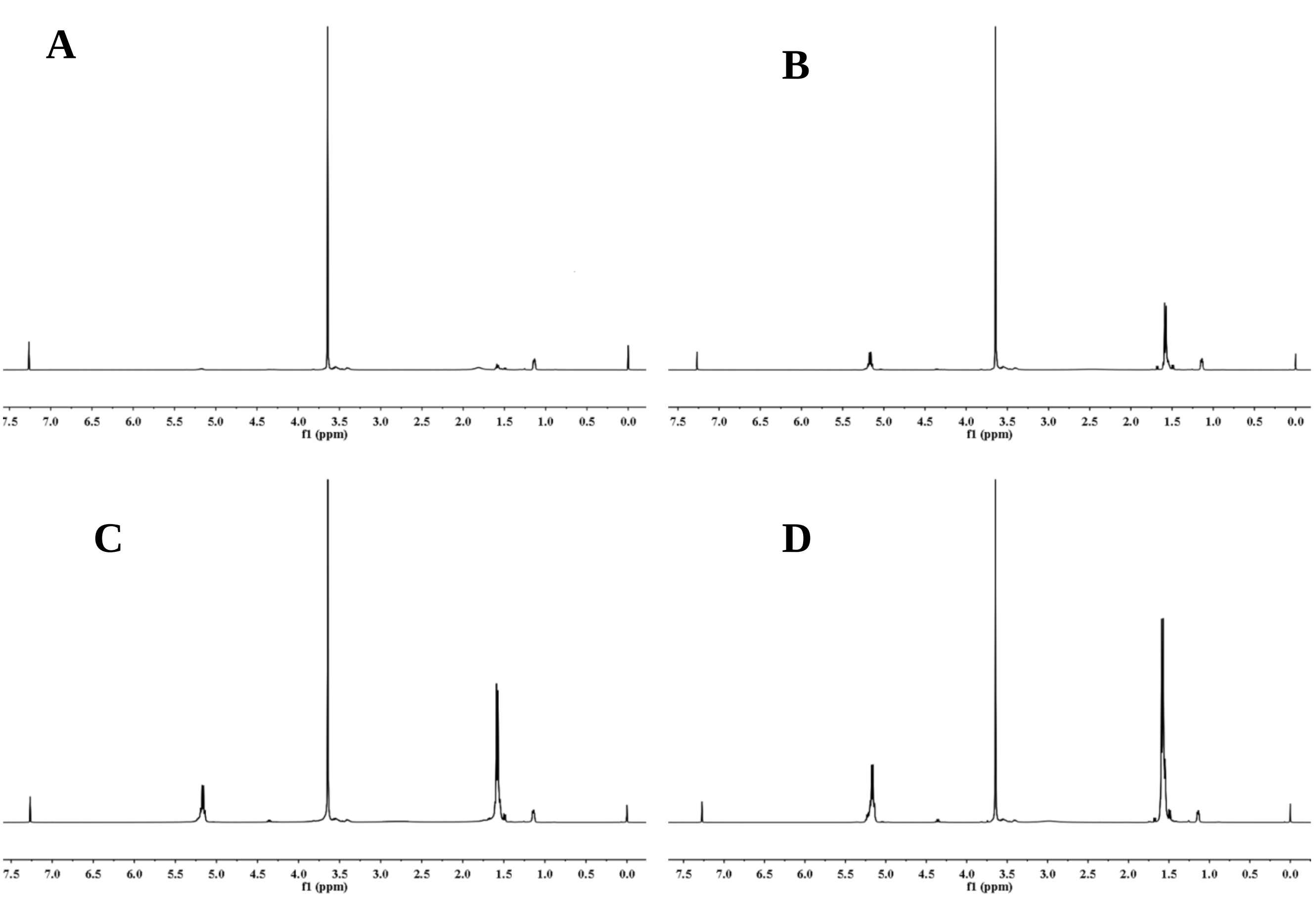

Figure S2. ^{1}H-NMR of PLFL copolymer $PLFL_{10K}$ (A), $PLFL_{20K}$ (B), $PLFL_{30K}$ (C), $PLFL_{45K}$ (D).

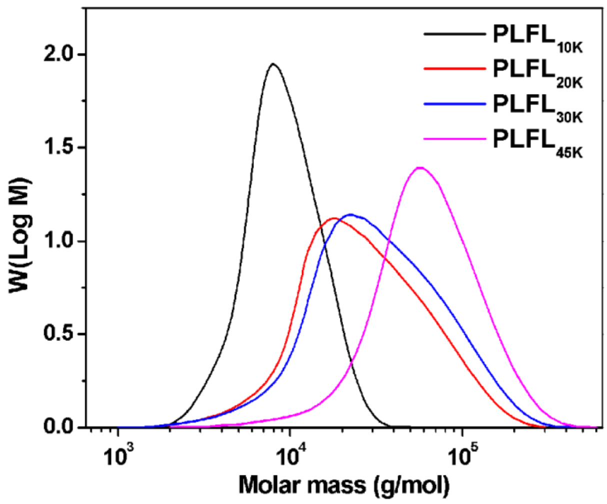

Figure S3. The GPC curves of PLFL block copolymers.

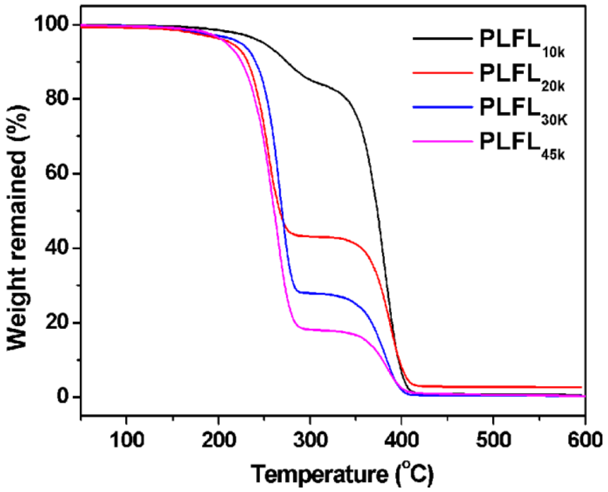

Figure S4. The TG curves of PLFL block copolymers.

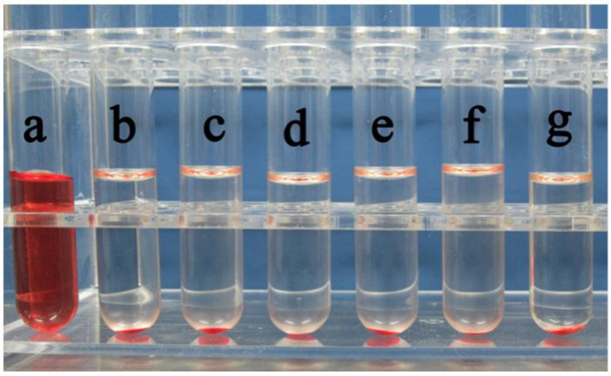

Figure S5. Hemolytic test on the blank PLFL nanofibrous MSs. The concentration of blank PLFL nanofibrous MSs is (a) Distilled water used as positive control; (b) Normal saline used as negative control; (c) 4 mg/ml;(d) 8 mg/ml; (e) 16 mg/ml; (f) 32 mg/ml; (g) 64 mg/ml.

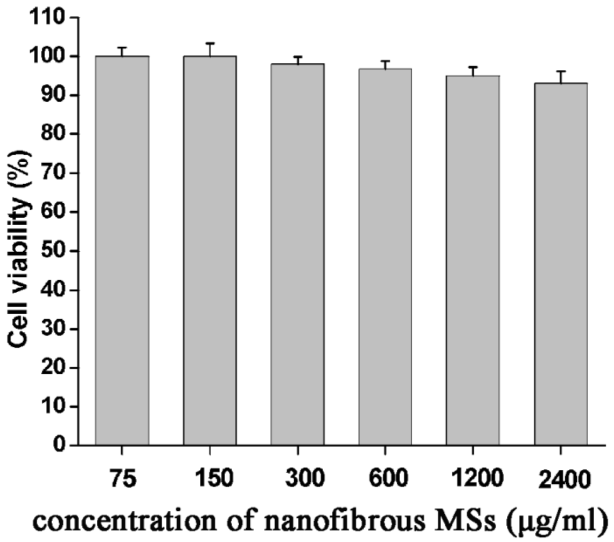

Figure S6. Cytotoxicity of blank PLFL nanofibrous MSs on L929 cells

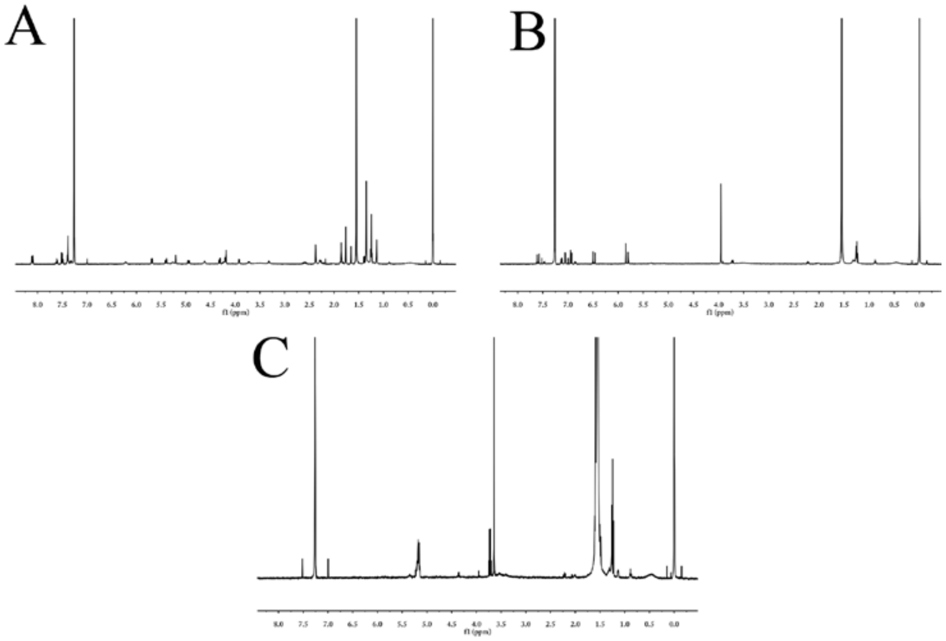

Figure S7. ^{1}H-NMR of DOC (A), CUR (B), DOC+CUR/nanofibrous microspheres (C).

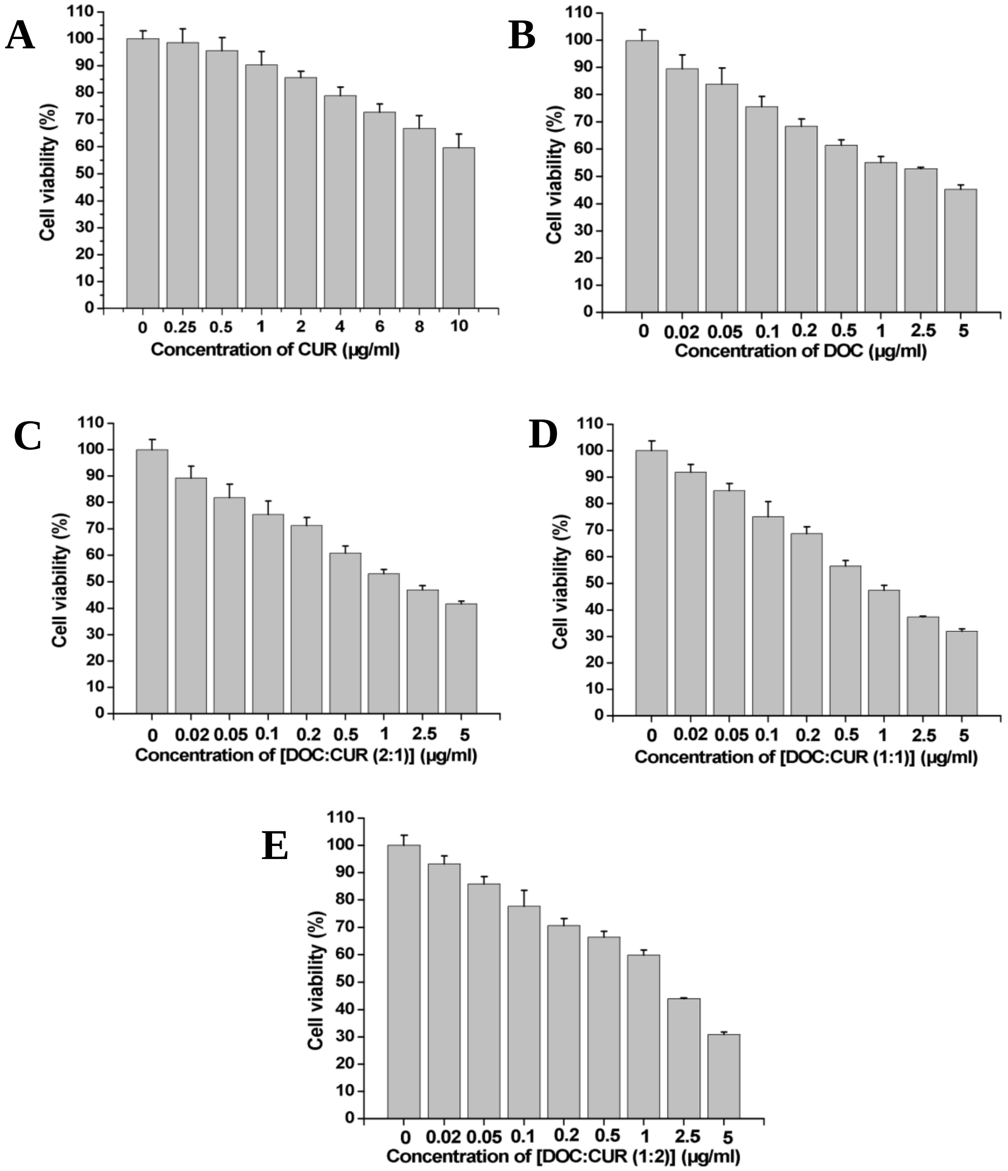

Figure S8. The MTT of free DOC (A), free CUR (B), free (DOC+CUR) (2:1) (C), free (DOC+CUR) (1:1) (D), free (DOC+CUR) (1:2) (E), which containing equivalent concentration of DOC. In Figure S8 (C-E), the horizontal ordinate refers to the doses of DOC.

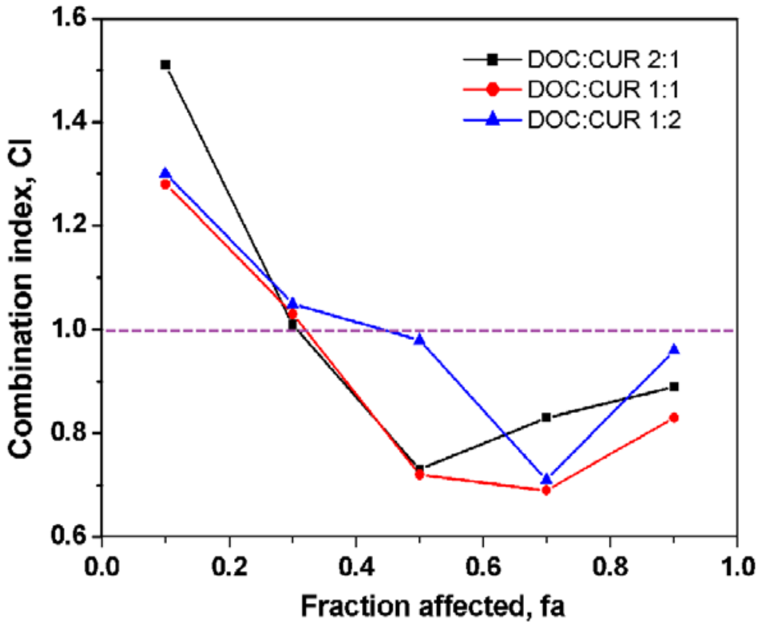

Figure S9. Combination Index (CI) for CT26 cells evaluated using the Chou and Talalay method. CI is plotted as a function of the fraction affected (fa), which represents the percentage of growth inhibition, evaluated using the MTT assay (0.5=50%). In this manner, the combination of DOC and CUR could be analyzed for synergism (CI <1) or antagonism (CI > 1). The straight line at CI =1 represents the additive effects of both drugs.
